# Usefulness of hospital emergency department records to explore access to injury care in Nepal

**DOI:** 10.1186/s12245-016-0120-9

**Published:** 2016-07-18

**Authors:** Santosh Bhatta, Puspa Raj Pant, Julie Mytton

**Affiliations:** Centre for Child and Adolescent Health, University of the West of England, Bristol, UK

**Keywords:** Emergency department, Unintentional injury, Hospital records, Nepal

## Abstract

**Background:**

Injuries are a major public health problem worldwide. Despite increasing morbidity and mortality from injuries in Nepal, it is not recognised in the government’s policy and programmes and few population-based studies have been published. This study describes the usefulness of hospital emergency department records to explore access to injury care in Nepal.

**Methods:**

A retrospective ED-based study was conducted at a governmental hospital in Nepal to review the routinely collected data for 1 year (1 January 2010 to 31 December 2010). The study was designed to provide cross-sectional data to describe the distribution of injuries by age, gender, ethnic group and injury mechanism.

**Results:**

Results showed that twice as many males as females attended the emergency department (14.6 vs. 7.0 per 1000), attendance varied by age with most (39.8 %) attendances in young adults of working age and over half of attendances were from just two ethnic groups (Brahmin (26 %) and Tamang (25.5 %). Road traffic injuries were the most common cause of injury (37.6 %).

**Conclusions:**

This study therefore showed the feasibility of using routinely collected hospital emergency department data to monitor injury inequalities in Nepal.

## Background

Injuries have become a major cause of premature deaths and disability throughout the world, with the burden of injury mortality and morbidity highest in low- and middle-income countries [1]. Nepal has one of the lowest income levels in the world [2]. The Global Burden of Disease study estimated that unintentional injuries accounted for 7 % (11,310) of all deaths occurring in the year 2010 in Nepal [3], a figure similar to the 8 % identified in reports by the Nepalese Ministry of Health and Population [4]. The Global Burden of Disease study estimated 10.4 million disability-adjusted life years (DALYs) due to all causes, of which over 7 % (744,000) DALYs attributed to unintentional injuries in 2010 [3].

The true burden of injury in Nepal is poorly understood. The number of studies on injuries in the country is small, and few have been published in international journals [5]. Often, injuries are considered to be the consequence of unavoidable accidents, and the prevention is rarely considered. However, as morbidity and mortality secondary to infectious diseases diminishes, the importance of injury as a major cause of illness, death and disability of all age groups of people is increasing. The rapidly changing economic, social and environmental landscape of Nepal, secondary to factors such as increasing motorisation, urbanisation, industrialisation, migration and changing life styles of people, may increase the prevalence of injury in the future [6].

In total, there are 65 districts and 10 zonal hospitals in Nepal [7], funded by the Nepalese Government and offering Essential Health Services (EHS), with basic services free of charge to poor, disadvantaged and indigenous groups [7]. Other services, or those not meeting the criteria for subsidised care, are required to pay. Many patients chose to attend the government-funded hospitals because of the subsidised cost of care. However, the facilities and services available in these hospitals vary, and some care is only available in the government hospital in Kathmandu or in private hospitals outside of the capital. The existing health system of Nepal does not offer ambulance services.

A study conducted in 11 hospitals across Nepal found that about 40,000 non-fatal injury cases were recorded in these hospitals during a period of 1 year (2008–2009) [8], with more than 80 % of these patients less than 45 years of age. A study conducted in Kathmandu in 2004 found that 32 % of the external causes of death among performed autopsies were due to unintentional injuries [9]. This data suggests that injury has an impact on the economically active population of Nepal. Road traffic injuries (RTIs) are the major cause of both fatal and non-fatal injuries among those who attend hospital [9–11]. In these studies, other frequent causes of injuries were occupational injuries, burns, violence, fall-related injuries and drowning. Injury appears to be an increasingly significant public health problem in Nepal, yet there is a relative lack of information available.

There is limited knowledge on whether injuries occur equally across the community [12]. There are a limited number of sources of routinely collected injury data in Nepal (e.g. there is no injury surveillance system and no robust death registration system). Emergency departments of the government hospitals provide initial assessment and decide whether to discharge, admit or refer the patient after basic treatment. The information from patients and hospital services is recorded manually, and there is no standardised data collection system. Therefore, this study explored the utility of using hospital emergency department records to understand the distribution of injuries by age, gender and ethnic group with a view to informing future research and data collection.

## Methods

### Study design

A 1-year retrospective review of the emergency department records of one government hospital in Nepal was conducted. The study was designed to provide cross-sectional data to describe the distribution of injuries by age, gender, ethnic group and injury mechanism. The study was conducted for a Masters in Environmental Health degree at the University of the West of England, Bristol, UK. Ethical approval was obtained from the Internal Review Committee (IRC) of the Kathmandu University Medical School and the Faculty Ethics Committee of the University of the West of England. The hospital administration allowed access to the medical records.

### Study setting

**Fig. 1:**
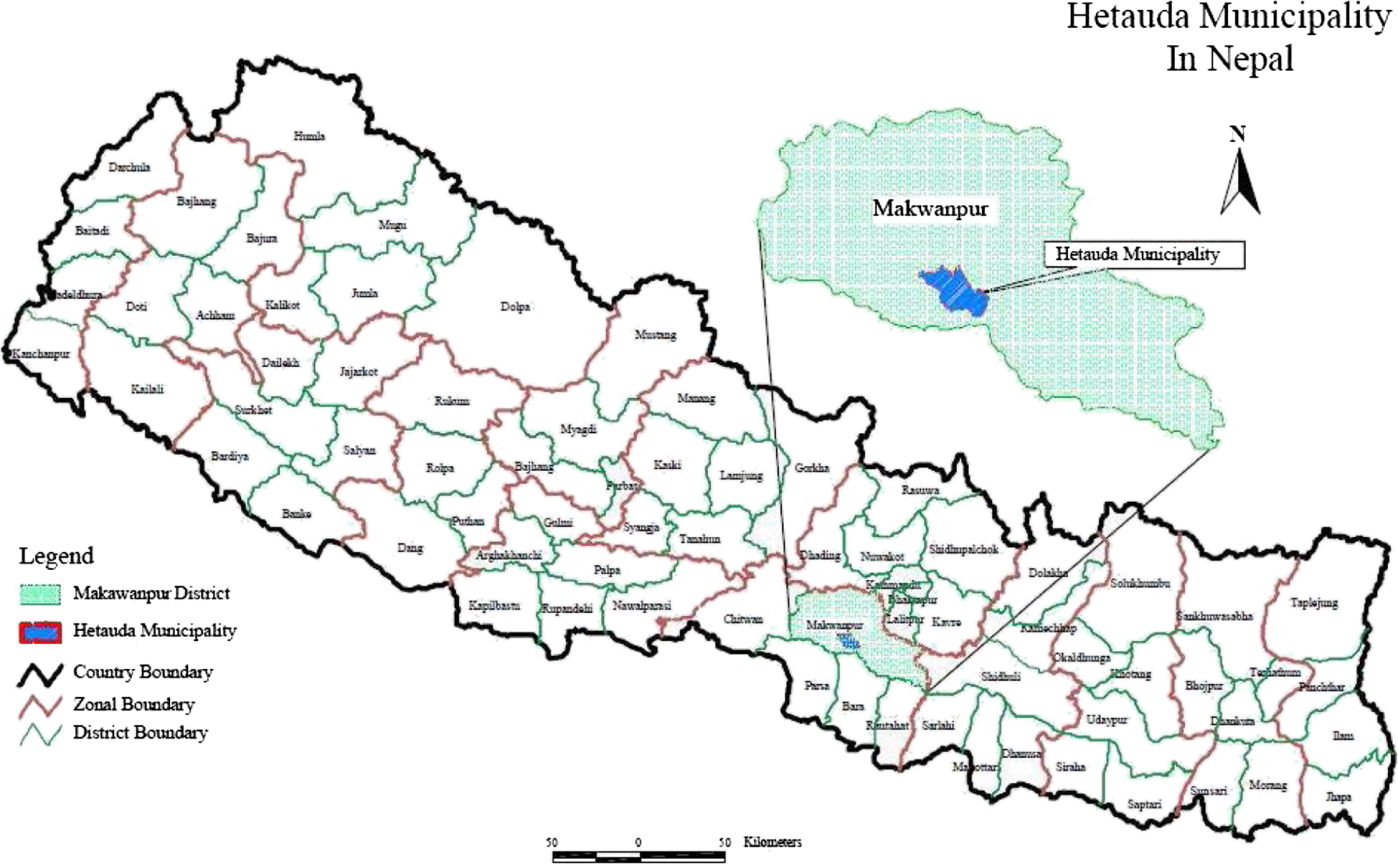
Map of Nepal showing Makwanpur and Hetauda. Source: Initial Environmental Examination (IEE) Report Sanitation and Sewerage Work Sub-Project [16]

The hospital studied was in Hetauda, an industrial city of Makwanpur district approximately 120 km (3-h drive) south-east of Kathmandu (Fig. 1). The district has an area of 2426 km^2^, making up 1.6 % of the total land area of Nepal. Hetauda is located at the country’s major highway intersection, between the east-west (Mahendra) and north-south (Tribhuvan) highways [13].

This district contains 78 different ethnic groups with their own languages and cultures. The major ethnicities are Tamang (indigenous), Brahmin, Chhetri, Magar, and marginalised groups such as Praja/Chepang, and endangered groups such as Bankariya [14]. There are altogether 36 village development committees (VDCs) and 2 municipalities in Makwanpur (www.ddcmakwanpur.gov.np). Hetauda is a municipality and the district headquarters of Makwanpur (Fig. 1).

Hetauda hospital is a 50-bed, government-funded hospital [13] which serves a population mainly living in the eastern and northern parts of the district (source: verbal inquiry with the Acting in-charge (paramedic) of the Emergency Department of the Hospital). Previously, the only hospital in the district, another community (eye) hospital, has recently been opened in Hetauda [15]. The Hetauda hospital was chosen because it was thought to illustrate the injury caseload of a typical government hospital. Most local injury cases will attend this hospital because of the long distances to other major tertiary care hospitals and lack of appropriate and efficient transportation systems. People from the west and southern parts of Makwanpur district may access hospitals in neighbouring districts that are geographically closer than Hetauda. The hospital has the facilities to provide treatment for both major and minor trauma but was not included in a hospital-based study by the Nepal Health Research Council [8].

### Data collection

Data were collected from the manually completed, paper-based record completed by the paramedic staff in the emergency department for all medical, surgical or injury cases presenting to the hospital. Each person attending the emergency department for treatment is routinely asked a set of questions, responses to which were recorded in a register: full name, age, gender, address and the presenting problem. The record also included the treatment given and information on whether the person was admitted to the hospital or went home. The information on the mechanism of injuries recorded on registers was used to identify the injury cases, and all unintentional injury cases were selected for this study. A data collection form was developed and piloted on 50 records prior to use. Data were hand-copied from the register onto data collection forms from the following fields: full name, age, gender, address, cause of injury, treatment and discharge (admission or home) for all patients who attended to the emergency department with injuries between 1 January 2010 and 31 December 2010. The address of the patients included the name of the VDC, ward number and locality. Towards the end of data collection, the first 10 cases of every fifth page of the data extraction form were cross-checked with the original source of data by a researcher (SB).

### Data coding

Injury data were coded after reading the details of the investigation and complaint mentioned in the register. Most cases of injuries recorded in the register were complete. The mechanism of injury was poorly specified for 275 patients (5.6 %) and missing for 17 patients. Any injuries where the mechanism of injury was missing or poorly specified were classified as ‘others’. The protocol used by the Nepal Health Research Council (NHRC) study was adapted for the purpose of coding the injuries [8]. We created additional categories for bites and for cuts and wounds because of their large numbers, and that they were easily identifiable from the ED register (Table 1).

**Table 1:** Case definition of cause category of unintentional injury (adapted from NHRC 2009)

Cause categories	Case definition
Road traffic injuries	Injuries resulted due to motorised or non-motorised vehicle crashes including cyclist and pedestrians
Bites	Animal bites such as dog bites, snake bites, and all insect bites including scorpion bites
Poisonings	All poisonings cases excluding food poisoning
Falls	Injuries resulting from falling on the same level or from different levels, including trees
Fire and burns	The burns/scalds and squeal of fires that resulted due to contact with flame, hot objects and water
Drowning	Injures resulted from emersion or submersion in the water
Cut wounds	Cutting and piercing or implants
Others	Injuries resulted due to environmental factors, machinery and electrical equipment and various other external causes were classified as 'others'

Ethnicities were identified according to the surname (caste) and grouped into the categories based on the information available in Central Bureau of Statistics [14]. In Nepal, there is a high correlation between a person’s surname and their ethnicity [17].

### Data analysis

Data were transcribed into MS-Excel spread sheets and then imported to SPSS v19.0 for analysis. Data were reported descriptively using frequencies and ranges and cross-tabulated to explore the distribution of injuries by age, gender and ethnic group. Differences between groups were investigated using non-parametric tests (chi-square). Observed data were compared to published data, where possible and appropriate.

## Results

A total of 4895 cases of unintentional injury were recorded attending Hetauda hospital between 1 January and 31 December 2010.

### Age and gender

Patients presenting with an injury were aged between 0 and 97 years. The mean age of study population was 26.9 (SD ± 18.2) years. Data on the age and gender of the patient were available for 97 % (4753/4895) of the study sample: 3238 (68.1 %) of attendees were men and 1515 (31.9 %) were women. Overall, men were twice as likely to attend with injuries as women (average male-to-female ratio was 2.1:1). However, the ratio varied by age group with the greatest difference being for adults aged 20–39 years where the male-to-female ratio increased up to 2.6:1 (Table 2).

**Table 2:** Distribution of population of Makwanpur district and injury patients attending ED at Hetauda Hospital, by age and gender

Age groups (years)	Population (estimated for 2005)^a^ (%)				ED patients (%)			
	Male	Female	Both	Gender ratio	Male	Female	Both	Gender ratio
0–4	31,429 (14.2)	29,859 (13.8)	61,288 (14.0)	1.1:1	226 (7.0)	165 (10.9)	391 (8.2)	1.4:1
5–19	80,048 (36.1)	78,716 (36.4)	158,764 (36.2)	1.0:1	1003 (31.0)	408 (26.9)	1411 (29.7)	2.5:1
20–39	65,419 (29.50)	65,428 (30.3)	130,847 (29.9)	1.0:1	1360 (42.0)	531 (35.0)	1891 (39.8)	2.6:1
40–59	31,987 (14.4)	29,723 (13.8)	61,710 (14.1)	1.1:1	480 (14.8)	262 (17.3)	742 (15.6)	1.8:1
60+	12,965 (5.8)	12,425 (5.7)	25,390 (5.8)	1.0:1	169 (5.2)	149 (9.8)	318 (6.7)	1.1:1
Total	221,848 (100)	216,151 (100)	437,999 (100)	1.0:1	3238 (100)	1515 (100)	4753 (100)	2.1:1

^a^Source: district profile of Makwanpur district [14]

The distribution of injury attendance by age category shows that the group most likely to attend the hospital with an injury were young adults of working age (20–39 years) representing 39.8 % of all injury cases (Table 2). The second highest proportion of ED visits (29.7 %) were found among the children aged 5–19 years. However, much smaller proportions of ED visits were for young children of age <4 years (8.2 %) and for people aged over 60 years (6.7 %).

Published data on the district profile of Makwanpur [13] indicate the proportion of the population by age category and are also shown in Table 2. These data suggest that the observed attendance at the ED with injury for older adult and over 60-year age groups are broadly in line with that expected from the population profile. However, the observed data suggest that the attendance at the ED with injuries is less common than might be expected for children aged 0–4 years and aged 5–19 years and more common than might be expected for young adults (20–39 years).

**Fig. 2:**
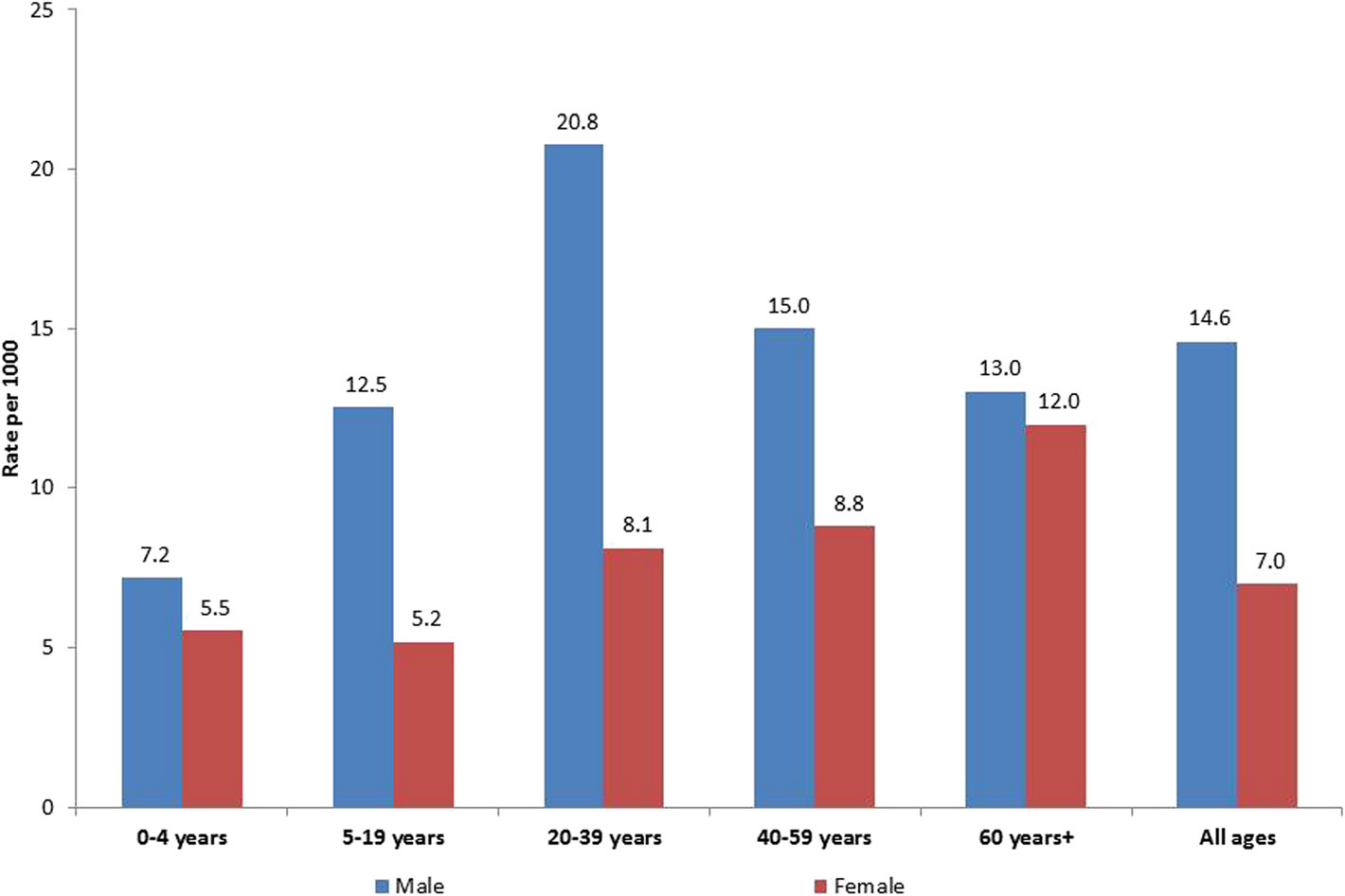
Rate of injury visits to Hetauda hospital by age and gender

To explore this further, we have used the data from the district profile of Makwanpur to estimate the rate of injury visits to the ED at Hetauda hospital by age and gender (Fig. 2). Overall, the ED visit rate for males in Makwanpur was 14.6 per 1000 population, compared to 7 per 1000 population for females. The rate of injury attendance for males varied by age group from 7.0 to 20.8 per 1000 population, whilst that for females ranged from 5.2 to 12.0 per 1000 population.

### Ethnicity

**Fig. 3:**
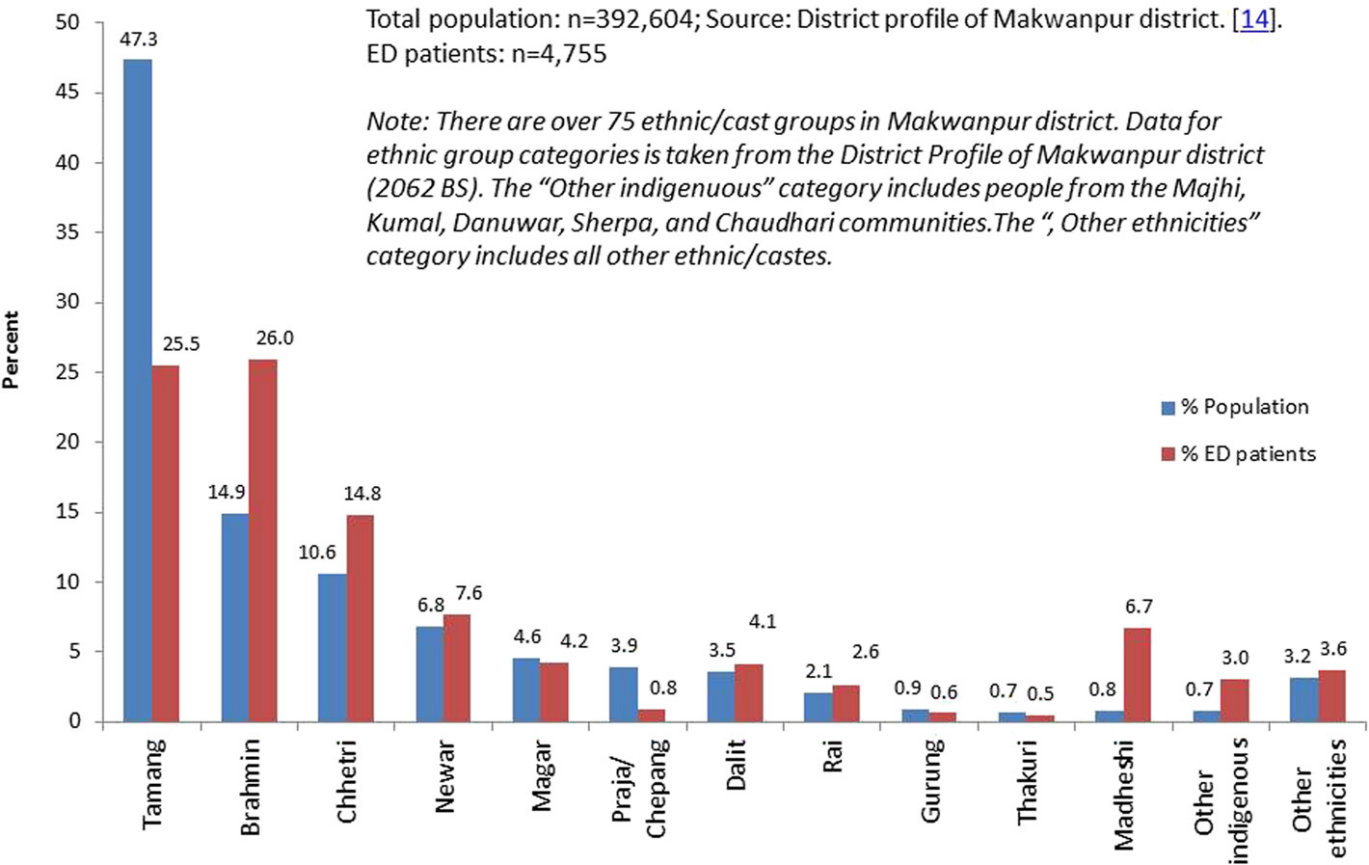
Distribution of injury attendances at Hetauda hospital by ethnic group compared to the distribution of ethnic groups in the total population. Total population: *n* = 392,604 (source: district profile of Makwanpur district) [14]. ED patients: *n* = 4755. Note: There are over 75 ethnic/cast groups in Makwanpur district. Data for ethnic group categories is taken from the district profile of Makwanpur district (2062 BS). The “other indigenuous” category includes people from the Majhi, Kumal, Danuwar, Sherpa and Chaudhari communities. The “the other ethnicities” category includes all other ethnic/castes

The ethnic group of injury patients attending the ED at Hetauda hospital was coded for 97.1 % (4755/4895) attendees. Patients came from a wide range of ethnic groups; more than half were Brahmin (26 %) and Tamang (25.5 %). Other common groups were Chhetri (14.8 %), Newar (7.6 %) and Madheshi (6.7 %). The data shows that the observed attendance differed from their expected by population estimates for several ethnic groups (Fig. 3).

The rate of ED injury attendance was estimated using the population size estimates from the district profile [14]. The rate varied widely from 2.5 per 1000 population in Praja/Chepang ethnic group to 97.7 per 1000 population in Madheshi ethnic group (Table 3 and Fig. 3).

**Table 3:** Rate of non-fatal injury related ED visits by ethnic groups, Makwanpur district

Ethnicities (*n* ^a^)	ED visits	Rate per 1000
Tamang (185,774)	1179	6.3
Brahmin (58,575)	1200	20.5
Chhetri (41,467)	684	16.5
Newar (26,764)	353	13.2
Magar (17,939)	193	10.8
Praja/Chepang (15,353)	39	2.5
Dalit (13,840)	190	13.7
Rai (8192)	122	14.9
Gurung (3541)	28	7.9
Thakuri (2636)	21	8.0
Madheshi (3164)	309	97.7
Other indigenous (2821)	138	48.9
Other ethnicities (12,438)	168	13.5
Total (392,604)	4624	11.8

^a^Census 2001 data. Source: District profile of Makwanpur district [14]

### Injury mechanisms

Data on the mechanism of injury was available for 96.8 % (4739/4895) of the study sample.

The mechanism of injury most commonly presenting to the ED was RTIs (37.6 %) and falls (29.8 %), followed by cuts/wounds (12.4 %) and bites (9.5 %). Burns and scalds, poisoning and drowning injuries were less frequently presented (Table 4).

**Table 4:** Distribution of injuries presenting to the ED by mechanism of injury and age category

Mechanisms	0–4 years (%)	5–19 years (%)	20–39 years (%)	40–59 years (%)	60+ years (%)	All ages (%)
RTIs	61 (15.7)	399 (28.3)	980 (51.9)	260 (35.3)	81 (25.6)	1781 (37.6)
Fall	167 (42.9)	524 (37.2)	339 (18.0)	230 (31.2)	152 (47.9)	1412 (29.8)
Fires and burns	25 (6.4)	21 (1.5)	26 (1.4)	6 (0.8)	2 (0.6)	80 (1.7)
Poisoning	28 (7.2)	5 (0.4)	3 (0.2)	1 (0.1)	0	37 (0.8)
Cut	52 (13.4)	209 (14.8)	211 (11.2)	93 (12.6)	23 (7.3)	588 (12.4)
Drowning	2 (0.5)	4 (0.3)	0	0	0	6 (0.1)
Bites	17 (4.4)	137 (9.7)	182 (9.6)	81 (11.0)	33 (10.4)	450 (9.5)
Others	37 (9.5)	109 (7.7)	147 (7.8)	66 (9.0)	26 (8.2)	385 (8.1)
Total	389 (100)	1408 (100)	1888 (100)	737 (100)	317 (100)	4739 (100)

The injury mechanisms varied by age. RTIs were the commonest cause of injury in adults 20–39 years (51.9 %) and 40–59 years (35.3 %). Adults (20–59 years) were statistically more likely to be injured in road traffic incidents than children (0–19 years) (chi-square = 211.09, *p* < 0.001) or older adults (60+ years) (chi-square = 53.77, *p* < 0.001). In contrast, falls were the commonest cause of injuries presenting to the ED for children aged 0–4 years (42.9 %) or 5–19 years (37.2 %) and for older adults aged 60+ years (47.9 %).

The mechanism of the injury varied by gender of the patient; Table 5 presents the ranked causes of injury by gender of patient. RTIs were observed as a leading cause of injuries (42.7 %) among males fallowed by falls (26.4 %). In contrast, falls were found to be the more predominant among females (38 %), followed by RTIs (24.7 %). Males were more likely to sustain road traffic injuries than females, and this difference was greater than would have occurred by chance (chi-square = 147.02, *p* < 0.001). In contrast, falls and bites were more likely to have occurred to women than men (*p* < 0.001).

**Table 5:** Ranked causes of injury in patients presenting to the ED, by gender

Rank	Male	Female	Total (both gender)
1	Road traffic injuries	Falls	Road traffic injuries
2	Falls	Road traffic injuries	Falls
3	Cut wounds	Bites	Cut wounds
4	Others	Cut wounds	Bites
5	Bites	Others	Others
6	Fire and burns	Fire and burns	Fire and burns
7	Poisoning	Poisoning	Poisoning
8	Drowning	Drowning	Drowning

## Discussion

### Strengths and limitations of the study

Whilst injury is increasingly recognised as an important cause of mortality and morbidity in Nepal, we believe this to be the first study to describe the utility of hospital emergency department records to explore inequality in injury in Nepal. We have shown that the use of hospital emergency services in Hetauda varies by age, gender and ethnic groups across a range of injury types. As there is only one government hospital in Makwanpur district, we believe that this data will have included the majority of emergency department-treated injuries in this locality. The study identified the complete record of every attendance at that one hospital over one calendar year and generated a large dataset of over 4700 records for analysis. Data extraction and coding were completed by one author (SB), reducing the likelihood of coding variations and errors.

This study has several limitations that should be acknowledged. The use of only hospital-based records means that this study was unable to identify people with injuries who had gone to private hospitals or hospitals outside of the district or to identify those who were injured but did not seek medical assistance. We have been unable to explore issues such as difficulty in access, or the distance from the hospital, or whether there were fatal injuries that were never taken to the hospital.

Data missing for some fields in the registers could have affected the accuracy of the ED data analysed. Fortunately, the proportion of missing data in the ED registers in this hospital was small. The study was not able to validate the injuries recorded in the register with any other source of information (e.g. records of patients admitted to hospital) in the time available. The decision to classify an ED attendance as due to ‘an injury’ was a subjective one, made by the on-duty-paramedics. The data recorded in this study is limited to the study site and therefore may not be generalisable to other parts of the country.

### Comparison of the findings with published literature

This study analysed over 4700 people attending the Emergency Department of Hetauda hospital in Makwanpur (Nepal) in the year 2010. We found that the majority of the patients were aged 20–39 years, followed by children aged 5 to 19 years. A similar pattern was reported in a study of ED data conducted by NHRC [8], and another study conducted in western Kenya also found that the proportion of ED patient was higher among 15–29- and 30–44-year age groups [18]. However, in contrast to the NHRC study, this analysis showed that only a small proportion of children aged 1–4 years were brought to the ED for injury-related treatment. The World Report on Child Injury Prevention [1] showed that young children are at high risk of injury. We are unable to determine the reason for the low rate of attendances in young children in this study. Reasons may include that they are not brought to hospital or that their injuries are more likely to result in death and, therefore, they do not reach hospital.

Patients attending the ED were more likely to be males than females for all age groups. This finding is consistent with other studies in Nepal [5, 8, 19]. A cross-sectional descriptive study of emergency department records from six major health institutions in Kathmandu and Bhaktapur of Nepal showed that the majority of patients (72.6 %) sustaining injuries were male [19]. This proportion is slightly higher than the finding of the present study. Our finding that the commonest age group to present with injuries were young adults may mean that these presentations are due to occupational injuries occurring to men. This shows that economically active population of Nepal may be more at risk of injuries, as has been reported in other low- and middle-income countries. The Global Burden of Disease study (2010) data also shows that the DALYs are the highest among the age groups 15–19, 20–24 and 25–29 years [3]. Because of rapid urbanisation and motorisation in low- middle-income countries, RTIs are also on the rise in these countries and burden on the poor people is higher [20, 21]. The World Report on Child Injury Prevention showed that burns and scalds were one type of injury more common in women and girls and largely associated with cooking and food preparation. There were relatively small numbers of burns and scalds identified in this dataset, and it is unclear why this is the case.

This study tried to explore injury inequality in terms of different ethnic groups attending the ED. Overall, the rate of the non-fatal injury varied considerably between ethnic groups from being the highest among the Medheshi (97.7 per 1000 population) to being the lowest among the Praja/Chepang, (2.5 per 1000 population), with the majority of the injuries occurring in Brahmin (26.5 %) and Tamang (25 %) groups. The reason for such variable attendance by ethnic group is unclear but could be related to the socio-economic condition, occupation and living environment of members of different ethnic groups. Variable access to hospital services and variable exposure to injury-risk situations may account for the differences seen. We are not aware that injury distribution by ethnic groups has been published in other studies conducted in Nepal. In Nepal, the ethnic groups are derived from castes of the people and this is routinely used as proxy indicator of socio-economic status [17]. Our study has recognised that there is inequality in seeking care for trauma (Fig. 3 and Table 3). One of the important aims of this paper was to explore whether there is inequality in ED attendances for trauma care.

This study revealed that more than one third of injury cases were due to RTIs and that the majority of victims of RTIs who visited the ED of study hospital were the age group of 20 to 39 years. The proportion of male patients due to the RTIs was noticed much higher as compared to female. This finding is consistent with the other hospital-based studies conducted in Nepal [5, 8, 11, 22]. Road traffic incidents are increasing in Nepal and are a common feature of the motorisation of low- and middle-income countries [6]. Roads in Nepal are often narrow with single lanes. The recent dramatic increase in the number of vehicles, together with the lack of strict traffic rules and regulations, lack of awareness about safe road use behaviour and road safety factors, and lack of regular vehicle maintenance, have been suggested as contributing to the problem [8, 19].

The second most common cause of injury was falls, as has been reported in the 2009 NHRC study. In our study, falls were found to be the leading cause of injury in female and accounted higher proportion than in males. In contrast, falls were the fifth causes for ED visits in western Kenya [18]. This finding differs from those reported in the 2009 NHRC study where the male population were mostly affected from all types of injury except poisoning. Our study shows that children aged <20 years and people over the age of 60 were more likely to be injured due to falls. The finding of the NHRC [8] has also shown that higher number of fall-related injury occurred among the children of age 5 to 14 years but did not report large numbers of falls in the elderly.

Injuries due to animal bites were most frequent in children (particularly girls) aged 5 to19 years and in young adults aged 20 to 39 years. The majority of patients sustaining bite injuries were bitten by scorpions. However, insect’s bites, dog and snake bites were also common causes of attendances at EDs. The mechanisms of injury in terms of cut and bites were not described in the study conducted by the 2009 NHRC. A similar study from Kenya found 10 % of the ED patients had cut injuries [18].

Very few injury cases attended the ED due to the poisoning or drowning in this sample; it is likely that some drowning cases were dead at the scene and were not transported to hospital. Similar to the other studies, our study revealed that injury due to drowning was less than 1 %, and males were more affected than females [8]. In this sample, there were only six drowning cases among children aged less than 19 years. Poisoning cases were also found to be more common in the male population than female and higher among the young children of age 0 to 4 years. This finding differs from other hospital-based studies, where females are more likely to be injured by the poisoning than male and poisoning cases were seen high among the adult [8, 23]. Few injury cases (<2 %) in this study were due to fires and burns. This finding is similar to another study conducted in Western Regional Hospital in Nepal [24]. A small proportion of injuries due to burns (3 %) were also reported in the 2009 NHRC study. We suspect that burn injuries do not present at the hospital because they are treated at home. This study has not explored whether the home treatment is satisfactory or what types of treatment are being given fire- and burn-related injuries. More males than females sustained injuries due to burns, and this finding is similar to the 2009 NHRC but contrasts with the results from another study [24].

Although this study has been able to report the frequency of injury types presenting to the ED, the injury records did not provide enough details to fully understand the cause, consequences and potential for prevention intervention.

### Implications

This study shows that hospital emergency department records are able to demonstrate inequality in injury occurrence by age, gender and ethnic groups. Exploration of the cause of the inequality and evidence of how to reduce those inequalities are beyond the scope of this article. High-quality information is necessary to know whether injury prevention interventions are making a difference. At present hospital, records are completed by hand, with variable information captured and without guidance to ensure that presentations are recorded consistently between hospitals. Such standardisation of ED records could form the basis for an injury-specific surveillance system across the country. A single digital system, developed using the rapidly developing IT infrastructure in Nepal, may mean that this initiative would not be an added burden to the paramedics working at the ED. Information about the costs of treatment could, in future, be used to illustrate the monetary burden of injuries to Nepalese society. Such a system would increase the usability of the data and aid the design of appropriate injury prevention interventions. In our study, particular groups (e.g. the very young, some injury types and some ethnic groups) do not appear to be attending ED as often as might be expected. If these groups did attend, this would place additional burdens on the resources and capacity of emergency departments.

## Conclusions

We know that injuries are an increasingly important cause of morbidity and mortality in Nepal. This study has demonstrated the utility of hospital emergency department records to explore inequalities in injury occurrence. With increasing motorisation and urbanisation of Nepal, it is likely that the incidence of RTIs will increase. The development of standardised hospital emergency department record systems would provide the potential to monitor changes in the incidence and cause of injury and to monitor inequalities in injury occurrence.
